# Number Needed to Scan: Evidence-Based Point-of-Care Ultrasound (POCUS)

**DOI:** 10.7759/cureus.17278

**Published:** 2021-08-18

**Authors:** Richard Amini, Asad E Patanwala, Hamid Shokoohi, Srikar Adhikari

**Affiliations:** 1 Department of Emergency Medicine, University of Arizona College of Medicine, Tucson, USA; 2 School of Pharmacy, University of Sydney, Royal Prince Alfred Hospital, Sydney, AUS; 3 Department of Emergency Medicine, Massachusetts General Hospital, Boston, USA

**Keywords:** number needed to treat, point-of-care-ultrasound, pocus (point of care ultrasound), research in emergency medicine, teaching in emergency medicine

## Abstract

Interest and enthusiasm, regarding the use of point-of-care ultrasound (POCUS), continues to grow among clinicians in multiple medical specialties. Ultrasound machines technology has advanced to allow for smaller, even handheld machines. Integration of automated imaging technology has made these machines more user-friendly. However, one of the concerns with the widespread availability of POCUS is the overuse and misuse of this technology. In order to maximize the clinical impact of POCUS, this manuscript seeks to discuss a novel concept called the “Number needed to scan” (NNS). The NNS is an expression of the number of POCUS examinations needed to be performed to attain a benefit to the patient or to prevent an adverse outcome of a procedure. NNS serves a dual purpose: it can help clinicians understand the magnitude of clinical impact when they apply POCUS, and it can help clinicians explain this magnitude in layman terms to their patients. In this manuscript, we have focused our NNS calculations on landmark articles in three major categories: change in management; safety and accuracy; and catching a missed diagnosis. As clinicians seek to be good stewards of POCUS, NNS should be a concept used to consider which patients will be most likely to benefit from a clinician performed ultrasound.

## Introduction

Interest in the use of point-of-care ultrasound (POCUS), continues to grow among clinicians in multiple medical specialties. Ultrasound technology has advanced to allow for smaller, even handheld machines. Furthermore, automated imaging technology has made these machines more user-friendly and has facilitated POCUS integration into residency training [1]. While there have been significant advances in ultrasound technology, and clinical adoption, the greatest clinical impact has been through the execution of robust evidence-based research for the use of POCUS in different diagnostic, prognostic applications, and procedural guidance [2-4]. These important studies have demonstrated the impact of POCUS on patient-centered and physician-centered outcomes [5]. Through these studies, clinicians have demonstrated a benchmark transition from “how good we are with performing POCUS” to “how effective POCUS is in different clinical applications.” In an effort to translate these POCUS effectiveness studies into real-world clinical scenarios, the authors have developed a concept called the “Number Needed to Scan (NNS).” NNS is similar to the well-established therapeutic concept, the number needed to treat (NNT). NNS is an expression of the number of POCUS examinations needed to be performed to change one management plant, prevent one adverse outcome from a procedure, or catch one missed diagnosis. The NNS is suggested as a measure of the effectiveness of POCUS and can be used for assessing the efficacy of performing a POCUS study or ultrasound-guided/assisted procedure.

POCUS is unique in that it affords clinicians and patients a diagnostic imaging choice that can be completed rapidly to answer specific questions while being free of radiation, repeatable, and affordable. However affordable, a strong POCUS program will have a significant fiscal impact from billing and reimbursement, and it is imperative for clinicians to keep stewardship a top priority [6,7]. Clinicians need a way to measure and compare the benefits of various imaging modalities. In this manuscript, we have focused our NNS calculations on landmark articles in three major categories: change in management; safety and accuracy; and catching a missed diagnosis. As clinicians seek to be good stewards of POCUS, NNS should be a concept used to consider which patients will be most likely to benefit from a clinician performed ultrasound. This manuscript will help optimize the integration of POCUS in the clinical setting, and provide a meaningful way to comparatively express the benefit or incorporate potential harms of performing POCUS as additional studies are published [8].

## Technical report

Number Needed to Scan

The number needed to scan (NNS) is an expression of the number of POCUS examinations needed to be performed to attain a benefit to the patient. The benefits are further defined as the change in management, improvement of procedural success, reduction of procedural complications, and a caught missed diagnosis. Defining these actual benefits may not be straightforward; as the use of POCUS can eliminate the need for a procedure, create the need for a procedure, or suggest an expedited consultation or surgical intervention. There are several elements that are required from the research literature to calculate the number needed to scan: the diagnosis in question or procedure/intervention; and measured benefits being achieved or events being prevented. The intervention is always constant, which is the application of POCUS. The NNS is more useful than a rate difference because it provides clinicians with a more concrete term of how much effort (how many ultrasounds) are needed to produce one outcome of interest. Furthermore, NNS is more useful than sensitivity and specificity alone, because it takes into consideration the day-to-day utility of clinical POCUS. This information can help guide clinicians when determining the benefits of taking time to perform a POCUS and evaluating the impact it can have on specific cases, diagnoses, interventions.

Because the expression NNT has been in clinical practice for over 30 years, it will be easy for clinicians to understand and adapt NNS into their daily practice. It is important to note that NNS is also similar to the NNT with regard to the magnitude of effect. Certain outcomes such as mortality or severe morbidity will alter what is considered a “good” NNS/NNT value. For example, in the medical literature, it is known that giving a patient an aspirin to prevent a myocardial infarction carries an NNT of 44 [9]. While this NNT number is large, the outcome is significant. In parallel, our study found that the NNS to prevent a post-thoracentesis pneumothorax requiring a chest tube is 63. While this too is a large number, the outcome is significant. While it is common knowledge that an NNT less than 10 is good and that an NNT of one is perfect, clinicians must look at the severity of disease, as life-altering outcomes will thus tolerate a higher NNT and NNS.

In this manuscript, the authors have chosen landmark POCUS studies in order to create examples of the NNS for various disease processes and procedures are discussed below. Similar to NNT, the true value of the NNS will be more robust when numerous studies and thus outcome measures can be combined through systematic reviews and meta-analysis.

How is NNS calculated?

Mathematically, the NNS is the reciprocal of the absolute risk reduction (ARR). The ARR in POCUS studies is the outcome measure difference calculated between the POCUS group and a control group (anatomic landmark, radiograph, clinical acumen). Applications of NNS include a change in management; safety and accuracy; and catching a missed diagnosis.

[NNS= 1/ARR]

Typically, the collective data in the systematic reviews are more likely to be used in defining the outcome of interest and those are the main sources for calculating NNS. Normally, the raw statistical outputs from these studies cannot be immediately applied to clinical practice. However, by using NNS we hope to find a simple method to facilitate immediate translation of data into clinical practice. Throughout this manuscript, the sentences containing summaries on NNS have been reworded in various ways to help clinicians understand and teach the concept as they apply NNS into their clinical toolbox. In order to best illustrate the concept of NNS, the authors have selected point-of-care ultrasound articles that focus on patient outcomes and evaluate the impact of POCUS on clinical practice. The NNS calculations demonstrated in this manuscript can easily be applied to future studies.

NNS to change management

NNS for Soft-tissue Infections

In 2005, researchers sought to evaluate the impact of POCUS for patients presenting to the emergency department (ED) with skin infections concerning cellulitis or abscess. They demonstrated that applying POCUS altered the overall ED management 56% of the time. These changes included canceling a decision to perform an incision and drainage (I&D) when one was shown not to be necessary on ultrasound, or proceeding with an I&D when one was shown to be necessary, but when a clinician had previously not intended to perform one [2]. Using this information, the ARR is 56%, and the number needed to scan to change management was two. Restated, an ED provider would need to scan two patients with soft tissue infections in order to alter the management in one case when compared to clinical assessment alone. In clinical scenarios where a clinician had made the decision to proceed with an I&D based on clinical assessment alone, POCUS results found no need for I&D and canceled the unnecessary procedure 36% of the time. In this situation, the ARR is 36%, and the number needed to scan in order to prevent an unnecessary and invasive incision and drainage is three [2]. Lastly, in patients determined to have an abscess requiring I&D by clinical assessment alone, POCUS determined the need for surgical consultation and advanced imaging 18% of the time. Using this information, the ARR is 18%, and the number needed to scan to discover a need for surgical consultation is six [2]. These findings were corroborated in a 2016 pediatric study on soft tissue infections in pediatric patients. In this study, clinicians were asked to evaluate soft tissue infections for the need of incision and drainage. where there was roughly a 24% rate of discordance between clinician examination and POCUS results [10]. Using this information, the ARR is 24%, and the number needed to scan to change the management plans in pediatric soft tissue infections was four. In conclusion, POCUS can significantly help clinicians in the evaluation of soft tissue infections and in the assessment of the need for I&D. Overall, POCUS can improve success rates and decrease complications rates in many cases of soft tissue infections. Based on the selected landmark studies, the NNS for soft tissue infections ranges from two to six depending on the outcome of interest.

NNS for Joint Effusions

In 2010, researchers sought to evaluate the impact of POCUS for patients presenting to the ED with joint space swelling and pain. They demonstrated that applying POCUS altered the overall ED management 65% of the time. These changes included canceling a decision to perform an arthrocentesis when one was shown not to be necessary on ultrasound, or proceeding with an arthrocentesis when one was shown to be necessary, but when a clinician had previously not intended to perform one [3]. In this study, the ARR is 65%, and the overall number needed to scan to create a change in management is two. In other words, when encountering a patient with joint pain, erythema, and swelling; for every two musculoskeletal POCUS performed, one change in management plan will occur. In clinical scenarios where a clinician had made the decision to proceed with an arthrocentesis based on clinical assessment alone, POCUS results found no need for arthrocentesis and canceled the unnecessary procedure 53% of the time [3]. In this situation, the ARR is 53%, and the number needed to scan in order to prevent an unnecessary and invasive arthrocentesis is two. Lastly, in scenarios where a clinician had made the decision not to perform an arthrocentesis by clinical assessment alone, POCUS demonstrated a drainable joint effusion and changed the management plan to perform the arthrocentesis in 69% of the cases [3]. Using this information, the ARR is 69%, and the number needed to scan to discover a need for surgical consultation is one. In conclusion, POCUS can improve joint effusion diagnosis and management plans in patients who may require arthrocentesis. In fact, POCUS can make an impact in nearly every patient who may require an arthrocentesis. Based on the selected landmark studies, the NNS for patients with concern for joint effusions ranges from one to two depending on the outcome of interest.

NNS for Hypotension

In 2015, researchers sought to evaluate the impact of POCUS in patients presenting to the ED with undifferentiated hypotension. They demonstrated that by applying POCUS to undifferentiated hypotensive patients, physicians can alter management plans 28% of the time [3,11]. These changes included changing the treatment plan, changes in securing a definitive diagnosis, and changes in resuscitative fluids or medications. In this study population, the ARR is 28% and the overall NNS to create a change in management is four. For every four patients with trauma-induced hypotension, the application of POCUS will change the management plan one time, when compared to clinical assessment alone. Researchers noted that POCUS was able to alter the treatment plan (including changes in fluid resuscitation, vasoactive medications, or blood transfusions) 25% of the time [11]. The ARR is 25% and the NNS to change the treatment plan in trauma patients is four. Lastly, the use of POCUS increased the rate of definitive diagnosis by 12% as compared to clinical assessments alone. The ARR is 12% and the NNS to achieve a definitive diagnosis is eight. In conclusion, POCUS can improve the evaluation, diagnosis, and management of patients presenting to the ED as trauma patients. Based on the selected landmark studies, the NNS for the evaluation and management of trauma patients ranges from four to eight depending on the outcome of interest.

NNS for Trauma

In trauma patients, there is general acceptance in the use of the Focused Assessment with Sonography in Trauma (FAST) exam. Studies have demonstrated over the years that the incorporation of the FAST exam has nearly eradicated the necessity to perform diagnostic peritoneal lavage [12]. This study demonstrated that when FAST exams are performed in the setting of trauma, it can help change management plans in 33% of cases [12]. In this case, the ARR is 33%, and this equates to an NNS of three. For every three trauma patients who undergo a FAST exam, one patient’s management plan will be altered. In addition, the use of the FAST exam has been shown to reduce the rate of computed tomography (CT) scans in these cases by 13% [12]. In this patient population, the ARR is 13% and the NNS to reduce a single CT scan order is eight. For every eight patients presenting as trauma patients who receive a FAST exam, one CT scan will be eliminated. In conclusion, POCUS can improve the management of patients presenting to the ED as trauma patients while also reducing radiation exposure. Based on the selected landmark studies, the NNS for the evaluation and management of trauma patients ranges from three to eight depending on the outcome of interest.

NNS to improve safety and accuracy

NNS for Central Venous Access

In the 2015 Cochrane database review on the application of POCUS to the placement of central venous catheters, the study also noted a 12% improved overall success rate for central line placement when ultrasound was used as compared to anatomical landmark technique alone [5]. The ARR is 12% and thus an NNS of eight. For every eight patients undergoing central venous access placement with ultrasound guidance, one patient will benefit from successful placement when compared to the anatomical landmark guided approach. Although ultrasound guidance and anatomic landmark guidance techniques yield similar final attempt success rates, ultrasound guidance is noted to improve first attempt success by 57% [5]. For first-pass success, the ARR is 57% which equates to an NNS of two. For every two central venous catheters placed under ultrasound guidance, one will benefit from first-pass success when compared to anatomical landmark guidance. In the same study, POCUS was demonstrated to reduce the overall rate of total complications by 71% [5]. In this patient population, the number needed to scan to prevent complications associated with central lines was one. It can be interpreted that every time a central line is performed, the use of ultrasound can prevent complications when compared to anatomical landmark guidance. In conclusion, POCUS can help clinicians in central venous access overall success rates, first-pass success rates, and decrease complications rates in nearly all cases. NNS ranges from one to eight depending on the outcome of interest.

NNS for Thoracentesis

Thoracentesis is often performed in the hospital setting for a variety of reasons. This procedure does carry the risk of pneumothorax [13]. The presence of pneumothorax after a thoracentesis has been documented between 0 and 19%. In a 2010 systematic review and meta-analysis, authors pooled data and calculated that the pneumothorax rate following ultrasound-guided thoracenteses was 4% while thoracenteses using anatomical landmarks had a pneumothorax rate of 9.3% [13]. This is a risk reduction of 5.4% if ultrasound is used. In this group of patients, the NNS is 19 for POCUS to reduce the rate of post-procedural pneumothorax. For every 19 patients undergoing a thoracentesis, POCUS will help prevent one post-procedural pneumothorax to anatomical landmark guided thoracentesis alone. While the magnitude of effect appears small in this procedure; this study also noted post-procedural pneumothoraxes required a chest thoracostomy 30% [12] of the time and the NNS is 63. For every 63 thoracentesis performed with ultrasound, one post-procedure chest thoracotomy will be prevented. In conclusion, POCUS can help clinicians performing thoracenteses. POCUS can decrease thoracentesis-associated pneumothorax rates and thoracentesis-associated chest tube thoracostomies. While the NNS ranges from 19 to 63, the complications carry significant morbidity, depending on the outcome of interest.

NNS for Lumbar Puncture

In 2007, Nomura et al. evaluated the impact of point of care ultrasound on lumbar puncture success rates. They randomized patients to receive either an anatomic landmark technique or an ultrasound-assisted technique for lumbar punctures performed in the emergency department. The groups were similar in age and body habitus, and the study noted a 27% failure rate in the anatomic landmark group and a 4% fail rate in the ultrasound group [14]. This 23% failure rate difference (27% - 4%) demonstrates an NNS of four. When these authors analyzed the obese population alone they demonstrated a 57% failure rate in the anatomic landmark group and a 0% failure rate in the ultrasound group [14]. The ARR, or in this case, the 57% failure rate difference equates to an NNS of two in the obese population. For every two times a clinician performs a lumbar puncture on obese patients using ultrasound assistance, one obese patient will benefit as compared to landmark guided techniques alone. In another study from 2014, POCUS was noted to reduce the risk of complications during lumbar punctures when compared to the anatomical landmark approach by 19% [4]. The ARR is 19% and thus the NNS in this study is five. For every five patients who receive ultrasound-assisted lumbar punctures, one complication will be prevented when compared to the landmark technique alone. In conclusion, POCUS can improve lumbar puncture success rates in all ages and BMIs and decrease complications. The overall NNS values range from two to five depending on the outcome of interest.

NNS for Peritonsillar Abscess

Peritonsillar abscesses (PTA) can be difficult to diagnose and treat. Ultrasound has been shown to have very good diagnostic utility in this patient population. In 2012, Costantino et al. conducted a randomized controlled trial for PTAs where POCUS was compared to clinical assessment for the diagnosis and management of PTA. In this study, POCUS had a 100% diagnosis rate, whereas clinical assessment had a 64% diagnosis rate [15]. In this group of patients, the ARR is 36%, and thus the NNS is three for POCUS to improve a diagnosis of PTA. For every three patients who present with concern for PTA by history, POCUS will help diagnose one patient with PTA when compared to clinical assessment without imaging. In addition, POCUS guidance had a 100% procedural success rate whereas landmark guidance demonstrated a 50% procedural success rate [15]. The ARR in this procedure is 50%, and the NNS is two. For every two patients identified to have a PTA by diagnostic imaging, ultrasound guidance during the aspiration of a PTA will help one patient to have successful procedural aspiration. In conclusion, POCUS can improve peritonsillar abscess diagnosis rates and drainage success rates. The overall NNS values for POCUS and PTAs range from two to three depending on the outcome of interest.

NNS to catch a missed diagnosis

NNS for Pneumothorax Diagnosis

According to a Cochrane systematic review and meta-analysis conducted in 2020, ultrasound was compared to chest radiograph for the evaluation of pneumothorax (PTX) [16]. In this article, the authors conclude that in a patient population of 100 in which 30 patients have pneumothorax; ultrasound would miss only three of 30 (3/30 = 10%) pneumothorax whereas chest radiograph would miss 16 of 30 (16/30 = 53%) pneumothorax [16]. This is a risk reduction of 43% if ultrasound is used every time. In this group of patients, the NNS is two for POCUS to improve a diagnosis of PTX. For every two patients who present with concern for PTX by history, POCUS will help diagnose one patient with PTX when compared to imaging with chest radiograph alone. POCUS would catch one otherwise missed PTX for every two times it is applied. In conclusion, POCUS can minimize missed diagnoses when compared to chest radiograph alone. POCUS can make an impact in one patient having a PTX every two times POCUS is used in this patient population.

## Discussion

While ultrasound is radiation-free and inexpensive, physician stewardship of this resource is necessary. Organizations, such as the American College of Physicians (ACP), American College of Radiology (ACR), have joined together to create national campaigns, such as Choosing Wisely, in order to curb health care costs through resource stewardship. Our manuscript describes a novel term that can help clinicians understand where the application of POCUS can be most effective. The NNS focuses on translating outcome measures into understandable terms that can benefit clinicians.

There are several limitations to this study. While this document serves as a starting point for the development of the NNS concept, with a review of how to calculate the term for various studies; the goal of this manuscript is not to be fully inclusive of all current POCUS literature. As studies conducted in the field of POCUS continue to be increasingly outcome-focused, it is important to revise and update NNS values. Furthermore, as more systematic reviews with meta-analyses are completed, there will be more applications for NNS.

## Conclusions

As expertise in POCUS continues to expand, clinicians will benefit from an understanding of the concept number needed to scan. The NNS is an expression of the number of POCUS examinations needed to be performed to obtain a benefit to the patient through changes in management; improvements in safety and accuracy; and catching a missed diagnosis. NNS serves a dual purpose: it can help clinicians understand the magnitude of clinical impact when they apply POCUS, and it can help clinicians explain this magnitude in layman terms to their patients. As clinicians seek to be good stewards of POCUS, NNS should be a concept used to consider which patients will be most likely to benefit from a clinician performed ultrasound.
